# Association of natriuretic peptides and receptor activity with cardio-metabolic health at middle age

**DOI:** 10.1038/s41598-024-60677-4

**Published:** 2024-04-30

**Authors:** Timothy C. R. Prickett, Eric A. Espiner, John F. Pearson

**Affiliations:** 1 Departments of Medicine, University of Otago, Christchurch, PO Box 4345, Christchurch, 8140 New Zealand; 2 Biostatistics and Computational Biology Unit, University of Otago, Christchurch, New Zealand

**Keywords:** ANP, BNP, CNP, cGMP, NPR1, Causal mediation analysis, Biomarkers, Endocrinology, Risk factors

## Abstract

Natriuretic peptides (NP) have multiple actions benefitting cardiovascular and metabolic health. Although many of these are mediated by Guanylyl Cyclase (GC) receptors NPR1 and NPR2, their role and relative importance in vivo is unclear. The intracellular mediator of NPR1 and NPR2, cGMP, circulates in plasma and can be used to examine relationships between receptor activity and tissue responses targeted by NPs. Plasma cGMP was measured in 348 participants previously recruited in a multidisciplinary community study (CHALICE) at age 50 years at a single centre. Associations between bio-active NPs and bio-inactive aminoterminal products with cGMP, and of cGMP with tissue response, were analysed using linear regression. Mediation of associations by NPs was assessed by Causal Mediation Analysis (CMA). ANP’s contribution to cGMP far exceed those of other NPs. Modelling across three components (demographics, NPs and cardiovascular function) shows that ANP and CNP are independent and positive predictors of cGMP. Counter intuitively, findings from CMA imply that in specific tissues, NPR1 responds more to BNP stimulation than ANP. Collectively these findings align with longer tissue half-life of BNP, and direct further therapeutic interventions towards extending tissue activity of ANP and CNP.

## Introduction

Multiple lines of evidence show that natriuretic peptides, acting via Guanylyl Cyclase (GC) receptors NPR1 and NPR2, confer numerous benefits to cardiovascular and metabolic health^1^. Whereas the activity and functionality of these receptors can be assessed in experimental animals, and during contrived changes in circulating concentrations of natriuretic peptides (NP) in humans^2,3^, less is known of the relationships linking NPs with receptor activity in the basal (resting) state.

The intracellular mediator of membrane bound GC receptors, cGMP, can readily access the extracellular space^4^, circulates in plasma and has been used in associative studies to clarify links of receptor status with organ dysfunction^5,6^ and with NP concentrations in plasma. To our knowledge no study has examined the physiological relationships of each of the NPs (ANP, BNP and CNP products) with cGMP across NP sensitive target tissues in subjects without history of heart disease. This is relevant to our understanding of the protective roles of cGMP in vivo—an important issue given the endocrine (ANP and BNP) and paracrine (ANP, BNP, CNP) roles and their variable potency in eliciting increase in cGMP when delivered intravenously^7^. Clarifying these links is increasingly important in light of the alternative role of NPR3 activity^8^ and the complexity of circulating forms of BNP products as currently measured in plasma^9^.

Here we report associations of plasma cGMP with bioactive and inactive plasma concentrations of ANP, BNP and CNP products in relation to a range of cardio and vascular risk factors in the first wave of a longitudinal study^10^ of 348 subjects at age 50 years, all without history of heart disease. Further, using causal mediation analysis (CMA), the relative strength of each NP mediating the cGMP response of specific tissues has been calculated, providing a measure of GC receptor activity. As previously reported in these subjects, higher concentrations of ANP or BNP—within the normal range—are associated with healthier metabolic and cardiovascular status whereas the opposite is true of CNP products—higher concentrations associate with impaired function. This dichotomy provides the opportunity to dissect out the links of individual peptides with cGMP, and to determine contributions of cGMP to a range of tissue malfunctions emerging at middle age. Based on our previous work^7^ showing that small contrived increments of plasma ANP are much more effective than BNP in raising plasma cGMP, we hypothesised^11^ that ANP but not BNP will be independently associated with cGMP. Although plasma cGMP shows little or no response to large contrived increments in CNP^3^, the increasing evidence of CNP’s adaptive role in combatting myocardial stiffening^10,12,13^, prompted us to further postulate that CNP will independently predict cGMP in subjects aged 50 years^11^.

## Results

As previously reported for this population (348 subjects, 55% female), median concentrations (inter quartile range) of ANP (9.2 (7.1–12.4) pmol/L), BNP (3.8 (3.1–5.2) pmol/L) and CNP (0.28 (0.20–0.40) pmol/L) were within the normal range. Median cGMP was 3.4 (2.9–4.5) nmol/L and similar in females and males (3.7 (2.9–5.0) and 3.4 (2.9–4.2) nmol/L respectively.

**Figure 1:**
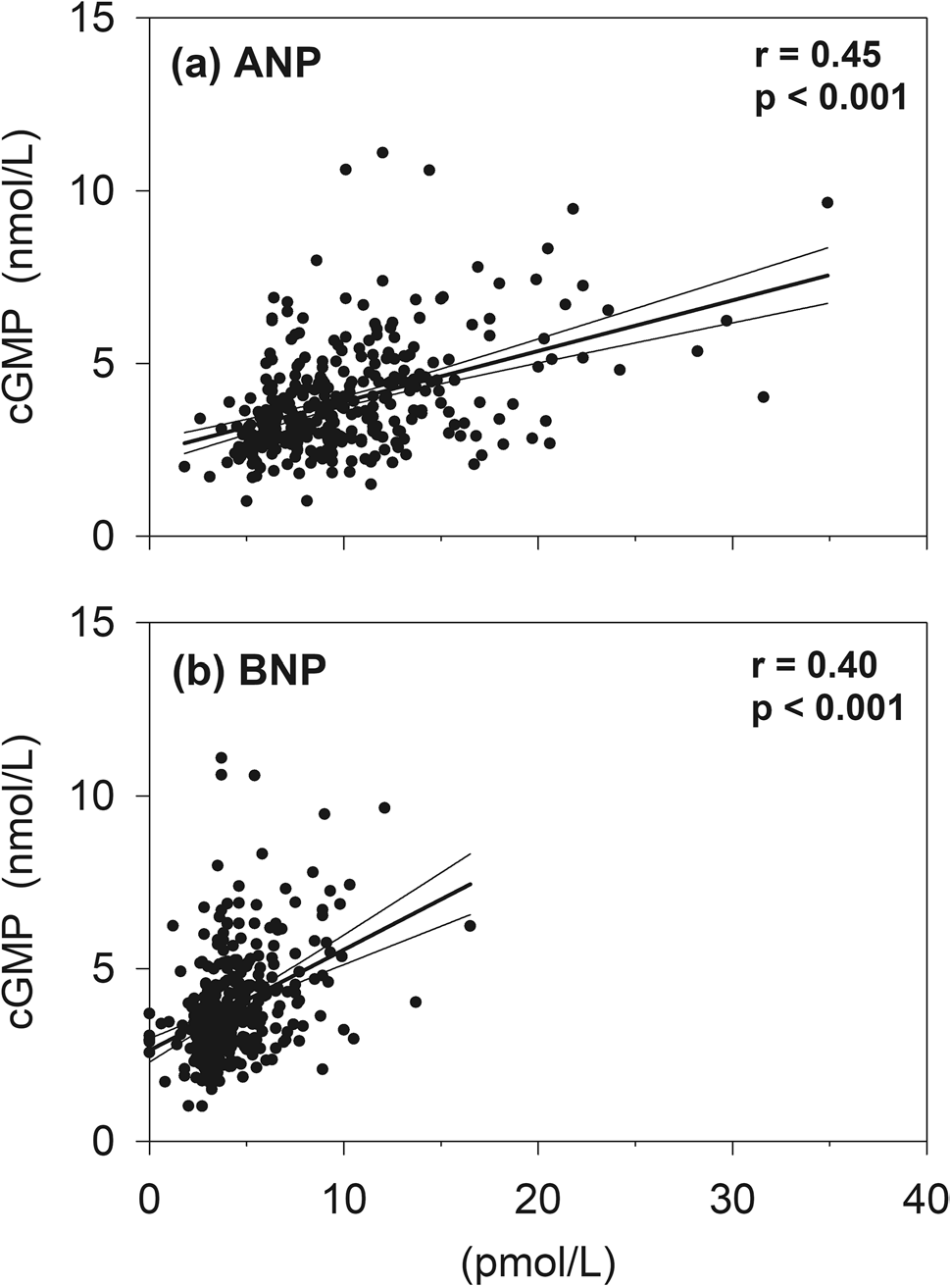
Correlations between circulating plasma concentrations of cGMP in healthy adults free of overt heart disease, aged 50 years, with ANP (**a**) or BNP (**b**). Regression line presented with 95% confidence interval.

Univariate associations of NPs with cGMP are shown in Table 1. Bio-active and bio-inactive forms of ANP and BNP were all positively associated with cGMP in both sexes. Conversely, associations with CNP and NTproCNP were weak and inverse. As shown in Fig. 1, although the associations of plasma concentration of ANP or BNP with concurrent cGMP were broadly similar, the association of plasma BNP with cGMP was confined to a reduced range and to much lower plasma concentrations of BNP when compared to associations with ANP.

**Table 1:** Associations of Natriuretic peptides with plasma cGMP.

	ANP	NTproANP	BNP	NTproBNP	CNP	NTproCNP
All (n = 352)	**0.45**	**0.50**	**0.40**	**0.38**	**− 0.14**	− 0.10
Female (n = 194)	**0.42**	**0.45**	**0.39**	**0.38**	− 0.10	− 0.10
Male (n = 158)	**0.46**	**0.52**	**0.36**	**0.30**	− 0.12	− 0.03

Boldface numerals indicate significant associations, p < 0.05.

### Associations of cGMP with cardio-metabolic health and role of NPs

There was strong evidence (all P < 0.01) that vascular risk factors, except eGFR, were negatively associated with cGMP after adjusting for sex and ethnicity, Table 2. The significant associations persisted after adjusting for ANP, BNP and CNP for all except for renin and LDL. CMA showed that the effect of cGMP on renin was partially mediated by ANP, BNP and CNP. Inverse association of HOMA with cGMP was reduced after adjusting for sex, ethnicity, and NPs but still significant. CMA provides evidence that this inhibition is partially mediated by BNP. The association of ANP and BNP products with vascular risk factors were similarly negative while the associations of CNP products were weakly positive (Supplementary Table S1).

**Table 2:** Associations of cGMP with vascular risk factors.

	cGMP by Sex and Ethnicity		Mediation		
	Without NPs	With NPs	ANP	BNP	CNP
Renin	**− 0.18**	− 0.09	**− 0.10**	**− 0.07**	**− 0.02**
BMI	**− 0.18**	**− 0.21**	0.04	0.00	− 0.01
Fat mass	**− 0.15**	**− 0.17**	0.02	0.00	− 0.01
HOMA	**− 0.23**	**− 0.19**	− 0.04	**− 0.07**	− 0.01
Triglycerides	**− 0.21**	**− 0.19**	0.00	− 0.04	0.00
LDL	**− 0.15**	− 0.11	− 0.02	− 0.04	− 0.01
Chol/HDL	**− 0.22**	**− 0.18**	− 0.01	− 0.04	− 0.01
eGFR	0.01	− 0.07			

Standardized regression coefficients for cGMP on vascular risk factors adjusted for Sex and Ethnicity with and without ANP, BNP and CNP. Mediation effect of ANP, BNP and CNP on the effect of cGMP on vascular risk factors adjusted for Sex and Ethnicity. Boldface numerals indicate associations with p < 0.05.

BMI, Body mass index; HOMA, Homeostatic Model Assessment for Insulin Resistance; LDL, low-density lipoprotein; Chol/HDL, total-cholesterol to high-density lipoprotein ratio; eGFR, estimated glomerular filtration rate.

There were 12 measures of cardiac function fitted to cGMP (Table 3). Heart rate adjusted for sex and ethnicity was significantly negatively associated with cGMP. CMA reveals that this effect was partially mediated by BNP (Table 3). There were 5 measures of cardiac function significantly positively associated with cGMP: LV stroke volume, LVESV, LVEDV, LA area and E/A ratio. Among these, there was evidence for partial mediation of LV stroke volume by BNP. The positive relationship of cGMP with LA area and E/A ratio was partially mediated by both ANP and BNP, and BNP respectively. Again, associations of ANP and BNP products were concordant with cGMP whereas CNP products were opposite to those of ANP and BNP (Supplementary Tables S2 and S3).

**Table 3:** Associations of cGMP with cardiac factors.

	cGMP by sex and ethnicity		Mediation		
	Without NPs	With NPs	ANP	BNP	CNP
Heart rate	**− 0.15**	− 0.09	− 0.06	**− 0.05**	− 0.01
Systolic blood pressure	0.06	0.04			
Diastolic blood pressure	0.01	− 0.02			
Arterial elastance	− 0.09	− 0.10			
LV elastance	− 0.06	− 0.03			
LV mass*	0.03	− 0.02			
LVEF	0.10	**0.14**			
LV stroke volume*	**0.15**	**0.15**	0.00	**0.03**	0.01
LVESV*	**0.12**	0.08	0.05	0.01	0.00
LVEDV*	**0.16**	**0.14**	0.02	0.03	0.01
LA area*	**0.13**	0.04	**0.07**	**0.05**	0.01
E/A	**0.16**	0.12	0.04	**0.07**	0.01

Standardized regression coefficients for cGMP on Cardiac risk factors adjusted for Sex and Ethnicity with and without ANP, BNP and CNP. Average Causal Mediation Effect of ANP, BNP and CNP on the associations of cGMP with cardiac risk factors adjusted for Sex and Ethnicity. Boldface numerals indicate associations with p < 0.05, * indexed to body surface area.

LV, left ventricle; LVEF, left ventricular ejection fraction; LVESV, left ventricular end-systolic volume; LVEDV, left ventricular end-diastolic volume; LA, left atrium; E/A, ratio of peak velocity blood flow from left ventricular relaxation in early diastole (the E wave) to peak velocity flow in late diastole caused by atrial contraction (the A wave).

### Multiple linear regression analyses

Modelling was performed on variables in components by demographics, natriuretic peptides, vascular risk factors and cardiac function (Table 4). There was no significant univariate difference in cGMP by sex or ethnicity, nor were sex or ethnicity significant in the final model. Demographics, NPs, vascular risk factors and cardiac parameters account for about 28% of the cGMP variation (p = 2.2 × 10^–12^ ANOVA; 14, 240 df). As shown in the component model, ANP, BNP and CNP explain about 19% of the variability in cGMP whereas vascular risk factors (10%) and cardiac parameters (9%) explain around 14% combined. Both ANP and BNP positively and independently predicted cGMP. In the complete model, ANP, CNP and LVESV are positively and independently associated with cGMP. Of the vascular risk factors, higher BMI and higher eGFR significantly and independently predicted lower cGMP (Table 4). There is evidence that vascular risk and cardiac function predict cGMP independently of NPs. When NT bio-inactive products are substituted for the bioactive NPs in the model very similar results are obtained except that BMI, eGFR and CNP are not significant independent predictors in the complete model (Supplementary Table S3).

**Table 4:** Demographic, Natriuretic peptide, Vascular risk and cardiac function effects on plasma cGMP.

	Component models				Complete model			
	*β*	*se*	*P*	*R*^*2*^	*β*	*se*	*P*	*R*^*2*^
**Demographic**				0.008				**0.280**
Sex (Male)	− 0.176	0.121	0.148		0.040	0.156	0.796	
Ethnicity (Māori)	0.017	0.179	0.924		0.131	0.160	0.413	
Ethnicity (Other)	− 0.088	0.267	0.741		0.034	0.241	0.888	
**Natriuretic peptide**				**0.194**				
ANP	0.338	0.074	** < 0.001**		0.338	0.075	** < 0.001**	
BNP	0.168	0.075	**0.025**		0.118	0.074	0.112	
CNP	0.106	0.063	0.095		0.152	0.063	**0.017**	
**Vascular risk**				**0.103**				
Renin	− 0.233	0.061	** < 0.001**		− 0.094	0.063	0.137	
BMI	− 0.209	0.064	**0.001**		− 0.236	0.113	**0.037**	
LDL	− 0.098	0.058	0.090		− 0.078	0.054	0.150	
eGFR	− 0.086	0.056	0.126		− 0.135	0.060	**0.027**	
**Cardiac function**				**0.089**				
LV Elastance*	0.248	0.105	**0.019**		0.158	0.097	0.104	
LVEF*	0.234	0.070	**0.001**		0.002	0.118	0.986	
LVESV*	0.404	0.114	** < 0.001**		0.242	0.112	**0.031**	
E/A	0.120	0.059	**0.043**		0.045	0.058	0.434	

Standardized coefficients *β*, standard errors *se*, *P* values and multiple *R*^*2*^ from component models: demographic, natriuretic peptides, vascular risk factors, cardiac risk factors, and the complete model with all terms from components. Skewed variables (ANP, BNP, CNP, renin, BMI, LDL and E/A) were log transformed. Boldface numerals indicate significance at P < 0.05, * indexed to body surface area.

BMI, body mass index; LDL, low-density lipoprotein; eGFR, estimated glomerular filtration rate; LV, left ventricle; LVEF, left ventricular ejection fraction; LVESV, left ventricular end-systolic volume; E/A, ratio of peak velocity blood flow from left ventricular relaxation in early diastole (the E wave) to peak velocity flow in late diastole caused by atrial contraction (the A wave).

## Discussion

Given the importance of cGMP’s role as the secondary messenger for NP signalling, it is surprising that there has been no comprehensive evaluation of NP, cGMP and organ function in people. In this first cross-sectional study of associations of all bioactive NPs with cGMP, and each of their associations with recognised tissues targeted by these endocrine and paracrine factors, we find ANP and BNP are each independently associated with plasma cGMP adjusted for CNP but not cardiac risk factors, (the NP component model, Table 4) in subjects age 50 years free of overt heart disease. Showing that BNP independently contributes to cGMP disproves our first hypothesis. Our second hypothesis that CNP will independently predict cGMP is sustained (Table 4, complete model)—however CNP’ s relationship with circulating cGMP is modest (β = 0.15) and has not been adjusted for vascular risk factors and cardiac function. Collectively the data shows that contributions of ANP to circulating cGMP far exceed those of other NPs—supporting the primacy of NPR1 signalling in maintaining cardiovascular and metabolic health at midlife. Counter intuitively, causal mediation analysis implies that in four of the six tissues examined where NP contributes to circulating cGMP, NPR1 responds more to BNP than ANP stimulation.

In studies linking NPs with cGMP, presumably the distribution, location, and abundance of functional natriuretic peptide guanyl cyclase receptors (NPR1 and NPR2), the local concentrations of bioactive NP accessing these receptors, and the relative affinity and potency of ligands stimulating cGMP are all relevant factors yet difficult to measure in humans. However, by constructing appropriate multiple linear regression models, a “global” measure of NP’s power to stimulate cGMP—relative to other factors can be obtained, adding to our understanding of their importance in maintaining cardiovascular homeostasis in well subjects studied in the basal state. Here we find that the strong positive associations of ANP and BNP, and the weaker inverse association of CNP, with cGMP align with the same dissociations previously identified between NPs and functional benefits at midlife^10^. Further, for the first-time causal mediation has been used to compare how the principal NPs contribute to GC receptor activity in specific tissues.

In previous studies using contrived changes in plasma NPs in healthy humans we showed that ANP is much more effective in raising plasma cGMP than is BNP or CNP^3,7^. The four-fold greater response of cGMP to ANP aligned with the higher affinity of ANP (eight-fold) compared to that of BNP for human NPR1^14^. These findings led us to hypothesise that ANP—but not BNP—would independently predict cGMP. The closely similar correlation of endogenous plasma ANP and BNP with cGMP in subjects at rest (Table 1 and Fig. 1) was therefore an unexpected finding, particularly in view of the much lower plasma concentration of BNP than ANP. Intact BNP(1–32) concentrations in plasma are likely to be > 30% lower than BNP levels measured in the current study^15^. As circulating ANP directly impacts vascular endothelial NPR1^1,16^, it is likely that ANP’s contribution to plasma cGMP will dominate and explain the findings from both exogenous and endogenous settings but in the later, paracrine actions—particularly from BNP and possibly CNP—could make important contributions, explaining the similar univariate associations shown in Table 1 and Fig. 1, and BNP’s significance as an independent driver of cGMP (Natriuretic peptide component model, Table 4).

Tissues chosen for associative studies examining contributions to plasma cGMP (Tables 2 and 3) were selected on the basis of their status as targets of NPs, and evidence of their strong association with concurrent concentrations of NPs (Supplementary Tables S1 and S2). As shown in Supplementary Table S4, adjustments were made for sex and ethnicity. Although there were significant differences in average NP and cGMP according to sex and ethnicity—cGMP lower in males, and NTproANP and NTproBNP lower in Māori and non-Europeans—there was no evidence that sex or ethnicity were independently associated with cGMP in the presence of NPs, vascular risk factors and cardiac parameters (Table 4). Of the eight vascular risk factors examined, all but one (eGFR) were significantly inversely associated with cGMP, and after adjusting for NPs, retained significance except for renin and LDL. Strong concordant inverse associations of cGMP and of ANP and BNP products with these vascular risks, and opposite (weak) associations of CNP (Supplementary Table S1) connect with findings in the adjusted analysis. CMA reveals that the significant inverse association of renin with cGMP was partially mediated by ANP, BNP and to a less extent by CNP. With respect to HOMA—a measure of insulin resistance—while data shows a persisting (but reduced) inverse association with cGMP after adjustment (Table 2)—CMA provides evidence supporting inhibition by BNP (Table 2). There is a wealth of previous data supporting inhibitory actions of endocrine ANP and BNP on renin secretion^1^, and on lipid accumulation^17,18^ via NPR1. That BNP, not ANP, mediates HOMA in people free of overt heart disease is surprising given the disparity in the circulating concentrations of the hormones. Paracrine production is unlikely to explain the findings as synthesis of ANP or BNP in peripheral tissues is thought to be insignificant^1^. However, in humans BNP is much less vulnerable to clearance by NPR3 and to degradation by NEP^19^—reflected by the much lower NTproNP/NP ratio (3, 38 and 56 for BNP, ANP and CNP products respectively in people with normal renal function) which likely prolong its activity in tissues such as adipocytes where both NEP and NPR3 are expressed^20,21^. Support for this is also provided by our earlier studies in humans where the rate of decline in plasma cGMP after withdrawal of steady state levels of BNP32 was much slower than found after withdrawal of ANP^2^. Differential handling of ANP and BNP locally may also underpin our earlier observations^7^ showing similar bioactivity (natriuresis, volume contraction, inhibition of basal renin and aldosterone) of these peptides evoked by much lower levels of BNP/cGMP stimulation. A further possibility favouring BNP activity relates to the negative feedback by sustained concentrations of ANP on NPR1^22^ which could dissipate ANP’s stimulation of cGMP at higher molar concentrations. Notably, the significant role of CNP in mediating renin inhibition by cGMP aligns with previous reports showing that CNP is expressed throughout the kidney, including the glomerulus and the mesangial tissues associated with the sites of renin synthesis^23^. As shown in Supplementary Table S1, CNP is positively associated with renin in both sexes whereas associations of ANP and BNP, and of cGMP, with renin are all inverse. Collectively this is the first evidence in humans of an important physiological (adaptive) role of CNP in maintaining cardiovascular homeostasis.

Participation of ANP and BNP via NPR1, and of CNP via NPR2 in regulating cardiac performance has been examined across 12 indices of cardiac function as shown in Table 3. The inverse association of cGMP with heart rate was partially mediated by BNP. Similar analysis of 5 other indices of myocardial function showed significant positive independent impact of cGMP on stroke volume, E/A ratio and LA area. All three were mediated by BNP—one of which (LA area) was also mediated by ANP. The dominance of BNP’s role in atrial and ventricular contractile function is not surprising in light of BNP’s paracrine function as evidenced by numerous studies in experimental animals and in humans (reviewed by Kuhn)^1^. The constitutive secretion of bioactive BNP—unlike atrial granules of stored ANP—could contribute to plasma cGMP without appreciable impact on peptide levels in the circulation. Again, its longer half-life in tissues, relative to ANP, could explain the findings. The confined range of the response curve to low BNP concentrations (Fig. 1) is also consistent with this speculation. The exception—LA area—(where ANP in part mediates NPR1 activity) is unsurprising in view of the much greater concentrations of ANP in atrial tissues which may explain the consistent association of ANP with LA area even in normal people^24^. No significant association was identified with CNP. Our hypothesis that CNP’s paracrine actions in myocardial tissues may impact plasma cGMP has not been fulfilled presumably because of the very low abundance (and faster clearance and degradation) in tissues of well people who have no history of heart disease. Once again, these directional associations align with the positive associations of measures of ANP and BNP with cardiac performance, and the weaker inverse association of CNP. The significant univariate association of CNP (inverse), and of ANP, BNP and cGMP (all positive), with E/A ratio in females (Supplementary Table S2) possibly relates to CNP’s adaptive response to impaired diastolic function resulting from myocardial fibrosis as reported recently in female mice^13^. Future follow up studies some 15 years later can be expected to reveal augmented NPR2 compensatory actions as age related stress accumulates^25^. Clarifying the respective roles and activity of NPs in vivo is highly relevant to implementing beneficial interventions such as those selectively augmenting cardiac CNP in experimental animal models with heart failure^26^, or ANP in human heart failure^27,28^. Our findings showing that BNP outperforms ANP in mediating specific tissue functions fit with these interventional studies which have harnessed the differential vulnerability among NPs to clearance and degradation.

Modelling by components (Table 4) reveals several new observations. Firstly, both ANP and BNP are positive independent predictors of cGMP, with NPs contributing to approximately 19% of the cGMP variance. Vascular risk (in our model, renin and BMI) and cardiac function (in our model LVESV, LVEF and LV elastance) combined account for 14% of the cGMP variance. Other drivers of cGMP unrelated to the natriuretic peptides include the nitric oxide (NO) pathway^29,30^ which in one interventional study accounted for some 30% of the change in the systemic venous plasma cGMP concentration^30^. It is also important to consider the possible role of phosphodiesterases (PDE) such as PDE9 which hydrolyses cGMP and is subject to regulation in pathophysiological settings such as heart disease and lipid disorders including obesity^31^. Inhibitors of PDE9 increase renin in animal models^32^, and supress lipogenesis in mice^31^, which suggests that PDE9 activity might contribute to the significant inverse associations of renin and BMI with cGMP (Table 4). Similarly, findings that measures of myocardial performance are positively related to cGMP (Table 4) connect with the beneficial actions of PDE9 inhibition on cardiac function in sheep with and without cardiac decompensation. In light of these connections, future study of the role of PDEs are clearly warranted in humans.

When all variables were combined (complete model), ANP (β = 0.338) and CNP (β = 0.152) were both independent predictors of cGMP, whereas cGMP is negatively impacted by eGFR and BMI. The loss of BNP predictive value in the combined model is likely to be due to the dominance of ANP’s actions on NPR1 in the vascular endothelium. On the other hand, the emergence of CNP’s impact on a quite different receptor (NPR2) comes to light presumably via multiple small contributions to circulating cGMP across a range of tissues, including CNP gene upregulation in the renal glomerulus, and secretions from the myocardial tissues as evidenced by associations of CNP with cardiac function (Supplementary Table S2). Although CNP has important actions in maintaining vascular integrity^33,34^ and in regulating blood pressure and flow in the microcirculation^35^, the largely sub endothelial location of NPR2, and the low abundance of bioactive CNP, likely account for the negligible impact of CNP on cGMP—relative to actions of ANP in the more accessible vascular endothelial tissues. Substituting bio-inactive NP forms for bio-active NPs did not alter the findings from modelling except CNP was no longer a significant predictor of cGMP in the complete model (Supplementary Table S3), and the inhibitory impact of BMI and eGFR were lost. Loss of BMI independence is consistent with the differential impact of bio-active versus bio-inactive forms of ANP and BNP (Supplementary Table S3). The aminoterminal NPs have a longer half-life and could in theory reflect gene expression and NP production in tissues across a wider time frame. However, the findings complement those derived from bioactive concentrations which represent real time relationships between concurrent plasma concentration and receptor response. Overall, the directional alignments of ANP/BNP/NPR1 associations across a wide range of vascular risks markers, opposite to those of CNP/NPR2 (Supplementary Table S1), and similar patterns identified in heart rate, stroke volume and sex specific associations with LVEDV and E/A (Supplementary Table S2), reinforce the dichotomy whereby higher values of ANP/BNP are beneficial to cardiometabolic health and those of CNP are adaptive^10^.

There are few previous studies of factors contributing to cGMP in people free of overt heart disease. In one large study^36^ of 1038 people in the community (mean age 68 years), in whom baseline cGMP was similar (4.7 nmol/L) to levels in the current study, plasma cGMP increased with age, was associated with higher blood pressure and lower eGFR, and was positively associated with NTproBNP (the only NP studied) in both sexes. Significant inverse associations of a range of risk factors reflecting lipid disorders and insulin resistance were identified. Modelling suggested that NTproBNP accounts for 9% of the cGMP variance. The authors attributed the positive link of NTproBNP with cGMP to adaptive (compensatory) responses to hypertension and increased myocardial wall stress though no measurements of cardiac performance were reported. Our smaller study differs in several important respects. Subjects were younger and all the same age (50 years). All were studied on the same site, had echo cardiographic markers measured together with in house specific and sensitive assays of all NPs (bioactive and inactive forms). Incorporating all three bioactive natriuretic peptides into multiple linear regression models, we find ANP, BNP and CNP combined accounts for 19% of cGMP variance measured in plasma. The significant negative impact of eGFR, after adjustment for other factors, aligns with the important role of the kidney in removing cGMP^37^. Unlike previous reports we found no correlation of cGMP with blood pressure in our younger cohort. Similar inverse univariate associations of lipids and other vascular risks with cGMP agree with those reported by Ying et al.^36^. In the course of preparing this manuscript for publication, Ma et al.^38^ reported the negative impact of angiotensin II on the associations of ANP or BNP with cGMP in 128 subjects from the community. In our study of 348 subjects, we also identified an inverse association of plasma renin activity with cGMP (unadjusted, r = − 0.20, p < 0.001, Supplementary Table S1). The fact that the association was lost after adjusting for NPs support the physiological role of NPs in constraining the renin angiotensin axis^1^.

### Strengths and limitations

All associations are based on circulating plasma cGMP concentrations and not on tissue concentrations which are likely to determine tissue responses. However, since studies from experimental animals^16^ show that NP-evoked plasma and lung tissue cGMP are strongly linked during proportionate changes in blood pressure, plausibly similar relationships between plasma and tissue cGMP apply in other well vascularised tissues. Variable rates of intra cellular degradation and extra cellular export of cGMP^39^ across tissues may affect levels accessing plasma—and have not been measured, and contributions from NO signalling to plasma cGMP have not been examined. The mediation analyses indicated sensitivity to effects such as these which would act as confounders. Since determining arterio-venous gradients of cGMP across organs in the basal state is unlikely to be informative^37^, the current approach of measuring co-associations of NPs and cGMP in plasma with markers of target tissue function is a valid and informative alternative. Strengths of the study include the wide range of biomarkers of cardio-metabolic health examined in a stable group of subjects, all the same age, without evidence of overt heart disease, and the measurement of all three natriuretic peptides in the same laboratory using highly sensitive and validated assays. The positive links of NPR1 activity with cardiometabolic health, and adaptive links of NPR2 activity in combatting early dysfunction, are significant new findings contributing to improved understanding of the mechanism underpinning cardiovascular health. As a baseline for a concurrent study in progress^11^ of the same population 15 years later, the study has the potential to determine causal relationships as people age, and enable beneficial interventions.

## Methods

### Study population

Details of the 348 subjects free of overt heart disease, recruited from 404 participants from the CHALICE study have been reported previously^10^. At the time of recruitment, 10.5% were receiving hypotensive therapy, and 9% were taking lipid lowering drugs. CHALICE is a multidisciplinary community study of participants aged 49–51 years recruited, with over-sampling from the Māori electoral roll^40^. Ethical approval for the CHALICE study was obtained from the Upper South A Regional Ethics Committee (URA/10/03/021) and conducted in accordance with the Declaration of Helsinki. Informed consent was obtained from all subjects and/or their legal guardian(s). All procedures were conducted at the University of Otago, Christchurch, and Canterbury District Health Board facilities by trained health professionals during the years 2010–13.

### Laboratory methods and procedures

Established humoral metabolic and cardiovascular risk factors were measured after an overnight fast using standardised procedures. Bio- and bio-inactive forms of ANP, BNP and CNP were measured as previously reported^41–43^ except that CNP was corrected for concurrent BNP cross reactivity^42^. Plasma cGMP was measured in baseline samples stored at minus 80 C. Plasma samples were extracted over C18 Sep-Pac columns (Waters Corp.) and assayed by radioimmunoassay as described previously^44^ except the primary antibody was sourced from Enzo Life Sciences (NY, USA, product code ADI-900-164). Intra- and inter-assay coefficient of variation for the cGMP assay at 4.1 pmol/L were 8.7% and 9.4% respectively. Standardized transthoracic echocardiography was performed using an iE33 ultrasound machine (Philips Life Healthcare) as detailed in Supplementary Information^10^.

### Statistics

Associations between plasma cGMP concentrations and humoral factors or clinical indices were analysed using linear regression models, adjusted where appropriate for variables known to affect cGMP (vascular, renal and cardiac function). All variables were assessed graphically and ANP, BNP, CNP, renin, BMI, LDL and E/A were log10-transformed to satisfy parametric assumptions. Effects of cGMP on cardio metabolic health, adjusted for sex (dichotomous) and ethnicity (prioritised ethnicity: Māori, Other, New Zealand European), were fitted with and without NPs. Any significant associations between cGMP and health factors were tested for mediation by ANP, BNP and CNP using the R package mediation^45^ by calculating Average Causal Mediation Effects, (ACME), with standard errors and p values calculated using 1000 simulations. Indirect effects, estimated as ACME, are reported in Tables 2 and 3. To quantify mediation, total and direct effects are reported in Supplementary Tables (Tables S5 and S6). Sensitivity analysis for all mediation models (maximum *ρ* < 0.3) indicated that there were likely to be additional unmeasured confounders other than sex and ethnicity which were included in all models.

Independence of effects on cGMP was assessed by multiple linear regression analysis in models that included variables from four components; demographics (sex and ethnicity), either bioactive NPs (ANP, BNP and CNP) or inactive NPs (NTproANP, NTproBNP and NTproCNP), vascular risk (Table 2) and cardiac function (Table 3). Variables were selected for their relevance to cGMP physiology/sites of production and removal. Stepwise selection of variables was used within each component to select a subset of variables which maximised fit (AIC). There was no evidence for any 2-way interactions. Modelling assumptions were graphically assessed and found to be adequately met and tested by ANOVA against the intercept only model. All tests were 2-sided and statistical significance was assumed when P < 0.05.

### Supplementary Information

Supplementary Tables.

## Data Availability

Restrictions apply to the availability of some of the data generated to preserve patient confidentiality. The corresponding author will on request detail the restrictions and any conditions under which access to some data may be provided.
