# Supplementary material for: Association of natriuretic peptides and receptor activity with cardio-metabolic health at middle age
Source: Sci Rep. 2024 Apr 30;14:9919. doi: 10.1038/s41598-024-60677-4 (PMC11061163; doi:10.1038/s41598-024-60677-4)
Supplement: Supplementary file 1 — Supplementary Tables. [file 41598_2024_60677_MOESM1_ESM.pdf]

Supplementary Information: Association of natriuretic peptides and receptor activity with cardio-metabolic health at middle age.

Timothy C. R. Prickett[1], Eric A. Espiner[1], John F. Pearson[1,2]

[1] Departments of Medicine, University of Otago, Christchurch, New Zealand.

[2] Biostatistics and Computational Biology Unit, University of Otago, Christchurch, New Zealand.

Supplementary Table S1 Associations of cGMP and Natriuretic peptides with vascular risk factors.

	cGMP	ANP	NTproANP	BNP	NTproBNP	CNP	NTproCNP
**Renin** – All	**-0.20**	**-0.26**	**-0.28**	**-0.24**	**-0.22**	**0.22**	**0.22**
– Female	**-0.18**	**-0.19**	**-0.29**	**-0.18**	**-0.28**	**0.23**	0.10
– Male	**-0.18**	**-0.29**	**-0.21**	**-0.25**	-0.10	**0.17**	**0.27**
**BMI** – All	**-0.16**	-0.01	**-0.18**	-0.06	**-0.13**	0.06	0.05
– Female	**-0.24**	-0.07	**-0.23**	-0.11	**-0.15**	0.11	0.11
– Male	-0.04	0.06	**-0.17**	-0.02	**-0.21**	0.01	0.03
**Waist** – All	**-0.20**	**-0.12**	**-0.26**	**-0.16**	**-0.24**	**0.12**	**0.17**
– Female	**-0.25**	-0.11	**-0.25**	**-0.15**	**-0.18**	0.10	0.13
– Male	-0.04	-0.01	-0.15	-0.02	**-0.19**	0.03	0.04
**HOMA** – All	**-0.23**	**-0.17**	**-0.37**	**-0.24**	**-0.32**	**0.17**	**0.14**
– Female	**-0.31**	**-0.21**	**-0.41**	**-0.27**	**-0.33**	**0.17**	**0.17**
– Male	-0.11	-0.12	**-0.32**	**-0.21**	**-0.35**	**0.17**	0.10
**TG** – All	**-0.24**	**-0.17**	**-0.33**	**-0.21**	**-0.29**	**0.10**	**0.22**
– Female	**-0.25**	-0.04	**-0.22**	-0.09	-0.15	0.07	**0.20**
– Male	**-0.17**	**-0.19**	**-0.35**	**-0.25**	**-0.32**	0.03	0.09
**Chol/HDL** – All	**-0.25**	**-0.20**	**-0.32**	**-0.23**	**-0.35**	**0.16**	**0.24**
– Female	**-0.27**	-0.11	**-0.22**	-0.13	**-0.18**	0.12	**0.17**
– Male	-0.16	-0.17	**-0.33**	**-0.21**	**-0.40**	0.09	0.13
**LDL** – All	**-0.17**	**-0.12**	**-0.15**	**-0.13**	**-0.22**	**0.11**	0.09
– Female	**-0.22**	-0.13	-0.10	-0.13	**-0.23**	**0.16**	0.11
– Male	-0.08	-0.07	**-0.18**	-0.07	**-0.17**	0.01	0.01

Boldface numerals indicate significant associations, $p<0.05$. Abbreviations: BMI, Body mass index; HOMA, Homeostatic Model Assessment for Insulin Resistance; LDL, low-density lipoprotein; Chol/HDL, total-cholesterol to high-density lipoprotein ratio; eGFR, estimated glomerular filtration rate.

Supplementary Table S2 Associations of cGMP and Natriuretic peptides with Cardiac function.

	cGMP	ANP	NTproANP	BNP	NTproBNP	CNP	NTproCNP
**Heart rate** – All	-0.11	-0.09	**-0.14**	-0.07	**-0.13**	0.03	-0.07
– Female	-0.17	**-0.21**	**-0.23**	**-0.18**	**-0.27**	0.11	-0.00
– Male	-0.13	-0.15	**-0.26**	**-0.17**	**-0.25**	0.08	0.11
**Arterial elastance *** – All	-0.05	0.04	**-0.14**	-0.10	-0.04	0.09	0.06
– Female	-0.07	0.02	**-0.20**	-0.05	-0.12	**0.17**	**0.22**
– Male	-0.12	-0.13	**-0.27**	-0.06	**-0.19**	0.14	0.16
**LV elastance *** – All	-0.02	0.01	-0.01	0.05	0.06	-0.01	0.01
– Female	-0.04	-0.01	0.05	0.01	0.05	0.07	0.15
– Male	-0.10	**-0.18**	**0.18**	-0.09	**-0.19**	0.08	0.12
**LVEF *** – All	**0.15**	0.10	**0.26**	**0.16**	**0.26**	**-0.16**	**-0.19**
– Female	0.13	0.02	**0.19**	0.09	0.15	-0.10	-0.06
– Male	-0.07	-0.07	0.12	0.03	0.12	-0.04	-0.01
**LV Stroke volume *** – All	0.10	-0.03	**0.21**	0.01	0.03	-0.07	-0.04
– Female	0.14	0.03	**0.26**	0.10	**0.18**	**-0.17**	**-0.24**
– Male	**0.19**	0.16	**0.25**	0.15	**0.22**	**-0.18**	**-0.24**
**LVESV *** – All	0.07	0.03	0.01	-0.03	-0.06	0.02	0.02
– Female	0.11	0.05	0.06	-0.01	-0.06	-0.05	**-0.16**
– Male	0.16	**0.24**	**0.18**	**0.18**	**0.21**	-0.10	-0.15
**LVEDV *** – All	0.10	-0.01	0.09	0.00	-0.01	-0.04	-0.02
– Female	0.15	0.04	**0.21**	0.07	0.10	-0.14	**-0.24**
– Male	**0.22**	**0.23**	**0.27**	**0.19**	**0.26**	**-0.17**	**-0.24**
**LA area *** – All	0.12	**0.20**	**0.31**	0.16	**0.21**	0.09	-0.11
– Female	0.15	**0.23**	**0.34**	**0.23**	**0.27**	-0.15	**-0.17**
– Male	0.10	**0.21**	**0.34**	0.09	**0.22**	-0.04	-0.10
**E/A** – All	**0.15**	**0.15**	**0.31**	**0.23**	**0.27**	**-0.15**	**-0.14**
– Female	**0.18**	**0.24**	**0.35**	**0.34**	**0.36**	**-0.19**	-0.14
– Male	0.13	0.04	**0.26**	0.08	**0.17**	-0.11	-0.14

*Indexed to body surface area. Boldface numerals indicate significant associations, $p<0.05$. Abbreviations: LV, left ventricle; LVEF, left ventricular ejection fraction; LVESV, left ventricular end-systolic volume; LVEDV, left ventricular end-diastolic volume; LA, Left atrium; E/A, ratio of peak velocity blood flow from left ventricular relaxation in early diastole (the E wave) to peak velocity flow in late diastole caused by atrial contraction (the A wave).

Supplementary Table S3 Demographic, bio inactive Natriuretic peptide, Vascular risk and cardiac function effects on plasma cGMP.

	**Component Model**				**Complete Model**			
	β	*se*	*P*	R^2	*β*	*se*	*P*	R^2
**Demographic**				0.016				**0.265**
Sex (Male)	-0.223	0.116	0.055		0.000	0.164	0.999	
Ethnicity (Māori)	0.039	0.175	0.826		0.208	0.161	0.199	
Ethnicity (Other)	-0.145	0.246	0.556		0.001	0.224	0.997	
**Natriuretic peptide**				**0.220**				
NTproANP	0.410	0.075	**<0.001**		0.359	0.081	**<0.001**	
NTproBNP	0.083	0.075	0.265		0.074	0.077	0.340	
NTproCNP	0.038	0.055	0.485		0.053	0.061	0.389	
**Vascular Risk**				**0.099**				
Renin	-0.221	0.058	**<0.001**		-0.085	0.060	0.157	
BMI	-0.196	0.062	**0.002**		-0.130	0.110	0.240	
LDL	-0.109	0.056	0.051		-0.066	0.053	0.213	
eGFR	-0.088	0.054	0.106		-0.079	0.062	0.199	
**Cardiac Function**				**0.085**				
LV Elastance*	0.218	0.102	**0.033**		0.164	0.094	0.082	
LVEF*	0.233	0.068	**0.001**		0.006	0.116	0.962	
LVESV*	0.375	0.108	**<0.001**		0.242	0.106	**0.023**	
E/A	0.115	0.057	**0.046**		0.002	0.056	0.976	

Standardized coefficients *β*, standard errors *se*, *P* values and multiple R^2 from component models: Demographic, Natriuretic Peptides, Vascular Risk Factors, Cardiac Risk Factors, and the complete model with all terms from components. Skewed variables were log transformed. Boldface numerals indicate significant at P<0.05, * indexed to body surface area. Abbreviations: BMI, body mass index; LDL, low-density lipoprotein; eGFR, estimated glomerular filtration rate; LV, left ventricle; LVEF, left ventricular ejection fraction; LVESV, left ventricular end-systolic volume; E/A, ratio of peak velocity blood flow from left ventricular relaxation in early diastole (the E wave) to peak velocity flow in late diastole caused by atrial contraction (the A wave).

Supplementary Table S4 Differences in average NP and cGMP levels by sex and ethnicity.

	Sex		Ethnicity		
	Male	P	Māori	Other	P
ANP	0.90 (0.86,0.93)	**<0.001**	0.96 (0.91,1.01)	0.94 (0.87,1.02)	0.103
BNP	0.90 (0.86,0.93)	**<0.001**	0.96 (0.91,1.01)	0.94 (0.87,1.02)	0.124
CNP	1.16 (1.09,1.23)	**<0.001**	1.04 (0.96,1.13)	1.01 (0.88,1.14)	0.622
NTproANP	0.91 (0.88,0.94)	**<0.001**	0.92 (0.88,0.96)	0.90 (0.84,0.97)	**<0.001**
NTproBNP	0.77 (0.73,0.82)	**<0.001**	0.92 (0.84,1.00)	0.83 (0.72,0.95)	**0.007**
NTproCNP	1.08 (1.06,1.11)	**<0.001**	1.01 (0.98,1.04)	1.02 (0.98,1.07)	0.518
cGMP	0.95 (0.92,0.98)	**0.004**	1.01 (0.96,1.05)	0.98 (0.91,1.05)	0.791

Coefficients with 95% Confidence intervals and P values (ANOVA) from linear regression of Log Natriuretic Peptides and cGMP on Sex and Ethnicity (European, Māori, Other). Boldface numerals indicate significant associations, $p<0.05$.

Supplementary Table S5 Mediation of cGMP on Vascular Risk by Natriuretic Peptides.

	ANP		BNP		CNP	
	Total	*Direct*	*Total*	*Direct*	*Total*	*Direct*
Renin	**-0.19** (-0.32,-0.07)	**-0.09** (-0.20,0.01)	**-0.19** (-0.31,-0.06)	-0.12 (-0.24,0.00)	**-0.19** (-0.32,-0.07)	**-0.17** (-0.30,-0.05)
BMI	**-0.19** (-0.31,-0.08)	**-0.23** (-0.36,-0.09)	**-0.19** (-0.30,-0.07)	**-0.19** (-0.31,-0.06)	**-0.19** (-0.31,-0.07)	**-0.18** (-0.30,-0.06)
Fat mass	**-0.16** (-0.26,-0.04)	**-0.18** (-0.30,-0.05)	**-0.16** (-0.26,-0.05)	**-0.15** (-0.27,-0.03)	**-0.16** (-0.26,-0.05)	**-0.15** (-0.25,-0.04)
HOMA	**-0.24** (-0.35,-0.13)	**-0.20** (-0.34,-0.07)	**-0.24** (-0.35,-0.14)	**-0.18** (-0.29,-0.06)	**-0.24** (-0.34,-0.13)	**-0.23** (-0.33,-0.12)
Triglycerides	**-0.22** (-0.32,-0.12)	**-0.22** (-0.34,-0.11)	**-0.22** (-0.31,-0.11)	**-0.18** (-0.28,-0.07)	**-0.22** (-0.31,-0.12)	**-0.21** (-0.31,-0.12)
LDL	**-0.13** (-0.23,-0.03)	**-0.11** (-0.23,0.00)	**-0.13** (-0.24,-0.02)	-0.09 (-0.23,0.06)	**-0.13** (-0.24,-0.02)	**-0.12** (-0.23,-0.02)
Chol/HDL	**-0.20** (-0.31,-0.11)	**-0.19** (-0.31,-0.08)	**-0.20** (-0.30,-0.11)	**-0.17** (-0.27,-0.05)	**-0.20** (-0.30,-0.10)	**-0.20** (-0.30,-0.10)
eGFR	-0.01 (-0.12,0.10)	-0.07 (-0.19,0.05)	-0.01 (-0.12,0.09)	-0.05 (-0.16,0.06)	-0.01 (-0.12,0.09)	-0.01 (-0.12,0.09)

Total and Direct Effect with 95% Confidence Intervals for mediation of cGMP on vascular risk by Natriuretic peptides.

Supplementary Table S6 Mediation of cGMP on Cardiac Factors by Natriuretic Peptides.

	ANP		BNP		CNP	
	Total	*Direct*	*Total*	*Direct*	*Total*	*Direct*
Heart Rate	**-0.15** (-0.27,-0.04)	-0.10 (-0.23,0.03)	**-0.15** (-0.26,-0.02)	-0.11 (-0.22,0.02)	**-0.15** (-0.26,-0.04)	**-0.15** (-0.26,-0.04)
Systolic Blood Pressure	0.07 (-0.05,0.18)	0.04 (-0.08,0.17)	0.07 (-0.05,0.18)	0.07 (-0.05,0.19)	0.07 (-0.04,0.19)	0.08 (-0.03,0.20)
Diastolic Blood Pressure	0.01 (-0.10,0.13)	-0.02 (-0.14,0.11)	0.01 (-0.10,0.12)	0.01 (-0.10,0.13)	0.01 (-0.11,0.11)	0.01 (-0.10,0.12)
Arterial Elastance	-0.08 (-0.21,0.05)	-0.09 (-0.23,0.05)	-0.08 (-0.20,0.05)	-0.07 (-0.20,0.07)	-0.08 (-0.21,0.06)	-0.07 (-0.20,0.07)
LV Elastance	-0.05 (-0.17,0.07)	-0.02 (-0.15,0.10)	-0.05 (-0.17,0.07)	-0.04 (-0.16,0.08)	-0.05 (-0.16,0.07)	-0.04 (-0.16,0.07)
LV mass*	0.03 (-0.10,0.17)	-0.01 (-0.13,0.12)	0.03 (-0.09,0.16)	0.00 (-0.13,0.15)	0.03 (-0.11,0.15)	0.03 (-0.11,0.16)
LVEF	0.11 (-0.02,0.23)	0.14 (-0.01,0.27)	0.11 (-0.01,0.25)	0.10 (-0.02,0.24)	0.11 (-0.01,0.23)	0.11 (-0.02,0.23)
LV stroke volume*	**0.15** (0.04,0.25)	**0.15** (0.03,0.27)	**0.15** (0.04,0.25)	**0.12** (0.00,0.23)	**0.15** (0.04,0.24)	**0.14** (0.04,0.23)
LVESV*	0.12 (-0.01,0.24)	0.07 (-0.07,0.21)	0.12 (0.00,0.23)	0.10 (-0.02,0.22)	0.12 (-0.01,0.24)	0.12 (-0.01,0.24)
LVEDV*	**0.16** (0.05,0.25)	**0.14** (0.02,0.24)	**0.16** (0.05,0.26)	**0.13** (0.02,0.24)	**0.16** (0.05,0.25)	**0.15** (0.04,0.25)
LA area*	0.13 (-0.02,0.26)	0.05 (-0.10,0.21)	0.13 (-0.01,0.26)	0.08 (-0.07,0.21)	0.13 (-0.01,0.27)	0.12 (-0.03,0.26)
E/A	**0.17** (0.05,0.29)	0.15 (0.03,0.27)	**0.17** (0.05,0.29)	0.12 (0.00,0.23)	**0.17** (0.04,0.29)	**0.14** (0.04,0.23)

Total and Direct Effect with 95% Confidence Intervals for mediation of cGMP on cardiac factors by Natriuretic peptides.
